# Screening of Microorganisms Isolated from Stingless Bees' Larval Food in the Biocontrol of *Meloidogyne incognita*

**DOI:** 10.2478/jofnem-2025-0028

**Published:** 2025-06-21

**Authors:** Guilherme Nunes Moreira Costa, Ana Carolina Costa Santos, Tamires dos Santos Paschoal, Anna Paula Martins Garcia, Anderson Rodrigues dos Santos, Carlos Ueira-Vieira

**Affiliations:** Laboratory of Genetics, Institute of Biotechnology, Federal University of Uberlândia, Uberlândia, Brazil; Faculty of Computer Science, Federal University of Uberlândia, Uberlândia, Brazil

**Keywords:** Bacteria, Biological Control, Management, *Meloidogyne incognita*, Nematicidal activity, Phytopathology, Plant-parasitic nematodes, Root-knot nematode

## Abstract

The plant-parasitic nematode *Meloidogyne incognita* causes significant financial losses in agriculture due to its wide range of host plants. Microbe-based biological control is increasingly being recognized as a viable and environmentally sustainable alternative to synthetic chemical nematicides. With this in mind, the present study evaluated the potential of microorganisms found in the larval food of stingless bees for the biological control of *M. incognita*. Screening of these microorganisms revealed the presence of a bacterium with nematicidal activity against *M. incognita*. Pot tests with soybean plants demonstrated that the bacterium Mq-MCK-07 reduced the population of *M. incognita*, while *in vitro* tests confirmed its nematicidal effect on second-stage juveniles (J2) as well as its inhibitory effect on egg hatching. DNA sequencing identified the bacterium as *Enterococcus faecalis* strain mandacaium, and revealed potential genes of interest for the control of multiple pathogens. This study provides a theoretical foundation for *E. faecalis* strain mandacaium as a promising eco-friendly nematicide.

Plant-parasitic nematodes (PPNs) infect a wide range of crop plants to obtain nutrients, thereby impairing the normal growth and development of their hosts (Ishida et al., 2020). Estimates suggest that the damage caused by these organisms can result in substantial financial losses, potentially exceeding US $100 billion annually worldwide (Abad et al., 2008; Elling, 2013; Coyne et al., 2018; Hu et al., 2022; Abdel-Rahman et al., 2023; Howland and Quintanilla, 2023). Among the various PPN species, root-knot nematodes (*Meloidogyne* spp.) have one of the broadest host ranges, and are capable of infecting numerous plant species, including vegetables, grasses, and even fruit trees (Blok et al., 2008; Desaeger et al., 2023; Nie et al., 2023; Schwarz et al., 2024; Tzean et al., 2024).

During parasitism, root-knot nematodes induce hyperplasia and hypertrophy in host cells, leading to the formation of thickened areas in the roots. These giant cells serve as feeding sites and disrupt the normal development of plant tissue (Trudgill and Blok, 2001; Almeida-Engler and Gheysen, 2013; Bartlem et al., 2014; Cabrera et al., 2015; Tzean et al., 2024). These swollen root areas, known as galls (or root knots), are the primary symptom of *Meloidogyne* spp. Infection, and give the genus its vernacular name (Jones et al., 2013). Gall formation damages the vascular system and impairs the plant's ability to absorb water and nutrients (Bartlem et al., 2014). *Meloidogyne incognita* is one of the most significant species within the genus due to its widespread distribution, short life cycle, high reproductive potential, and substantial economic impact. Additionally, infection by these nematodes can promote secondary infections by other pathogens such as fungi and bacteria, resulting in further damage and reductions in crop productivity (Jones et al., 2013; Gattoni et al., 2023).

Currently, several strategies are employed to control *Meloidogyne incognita* populations, with the most common approaches being the use of chemical nematicides and resistant cultivars (Giannakou and Anastasiadis, 2005; Jordan, 2018; Grabau et al., 2021; Ploeg et al., 2024). However, the excessive use of chemical nematicides poses risks to both the environment and human health, and can be expensive, particularly in areas with widespread nematode infestation (Starr et al., 2007; Aktar et al., 2009; Coyne et al., 2018; Bui and Desaeger, 2021). The use of resistant cultivars also has limited effect, as developing new cultivars involves lengthy selective breeding processes and is constrained by the limited availability of genetic resources conferring resistance to PPNs (Davies and Elling, 2015). Crop rotation can contribute to nematode control, but it is not always a practical solution due to the logistical and equipment challenges associated with managing diverse crops (Kirkpatrick and Sasser, 1984; Chen and Tsay, 2006). Consequently, alternative strategies that are both effective and environmentally sustainable, such as biological control, have received increasing attention (Bui and Desaeger, 2021; Hu et al., 2022; Tian et al., 2022; Mouniga et al., 2023).

Biocontrol methods have proven to be environmentally safe and effective strategies for controlling pests and pathogens, including PPNs (Mhatre et al., 2019; Lahlali et al., 2022). Recent studies have identified various microorganisms, such as fungi and bacteria, as potential agents for the biological control of PPNs (Silva et al., 2017; Jin et al., 2019; Migunova and Sasanelli, 2021; Nie et al., 2023; Alves et al., 2025). Some studies have described microorganisms with specific activity against *M. incognita* (Kim et al., 2018; Park et al., 2020; Pocurull et al., 2020; Sharma et al., 2020; Wang et al., 2022). Bacteria from the genera *Bacillus* and *Pseudomonas*, which are commonly found in the plant rhizosphere, play a prominent role in the biological control of PPNs (Mhatre et al., 2019).

Microorganisms used in biocontrol interact with PPNs in various ways. Some species parasitize eggs and juveniles, while others inhibit egg hatching or repel second-stage juveniles (J2) of root-knot nematodes (Davies and Curtis, 2011; Jin et al., 2017; Topalović et al., 2019; Cheng et al., 2017; Park et al., 2020; Khoja et al., 2021; Sun et al., 2021). Species from the genera *Pasteuria* and *Pochonia* exhibit multiple modes of action, including parasitism of nematode eggs through to adult females of *Meloidogyne* spp., but they can also secrete metabolites with nematicidal activity, or compounds that activate plant defense mechanisms against nematodes (Giné et al., 2016; Ghahremani et al., 2019).

Several microorganisms associated with stingless bee colonies appear to play a role in nectar fermentation and pollen maturation, as well as in the production of antimicrobial and nutritional compounds that benefit bees and protect the hive from pathogens (Dillon, 2004; Hamdi et al., 2011; Vásquez et al., 2012; Voulgari-Kokota et al., 2019). Recent studies have highlighted bacteria associated with stingless bee larval food as a promising source of probiotics, enzymes, and biocontrol agents against pathogens. Our group recently demonstrated that microorganisms associated with larval food from bees of the genera *Melipona* and *Tetragonisca* are capable of producing antimicrobial substances effective against both Gram-positive and Gram-negative pathogens (Santos et al., 2022). Therefore, the objective of this study was to evaluate the potential use of microorganisms isolated from stingless bee larval food for the biological control of *Meloidogyne incognita*.

## Material and Methods

*Obtention of microorganisms:* The fungi and bacteria used in this study were obtained from the Collection of Microorganisms Isolated from Stingless Bee Larval Food (CoMisBee) at the Laboratory of Genetics, Federal University of Uberlândia (Santos et al., 2023). Isolates were randomly picked up to initiate biotechnological prospecting. Whole-genome sequencing was performed only on isolates that exhibited nematicidal activity. Bacterial isolates were reactivated on BHI agar and incubated for 24 hours at 37 °C. A single colony was then transferred to 50 mL of Luria-Bertani (LB) medium and incubated at 37 °C ±1 for 48 hours with shaking at 200 rpm. To obtain the supernatant, cultures were centrifuged at 10,000 g for 4 minutes to pellet the bacterial cells. The supernatant was then separated and filtered through a 22-μm membrane. Fungal isolates were reactivated on potato dextrose agar (PDA) and incubated for 120 hours at 30 °C. After the formation of a spore mat, the surface was washed with 1 mL of 0.9% saline solution, and the spores were collected.

*Study location and environmental conditions:* The experiments with plants were conducted in a greenhouse at the Federal University of Uberlândia, Uberlândia, MG, Brazil, located at 18°53′04″S, 48°15′36″W, and 937 meters above sea level. The region is classified as Aw (tropical savanna) according to Köppen's climate classification system (de Sá-Junior et al., 2012). Greenhouse conditions were maintained at an average temperature of 28 ± 4 °C. All plants were irrigated once daily at 8:00 a.m. using an automated sprinkler system. The study was carried out between October 2023 and May 2024.

*Nematode inoculum:* The initial inoculum of *Meloidogyne incognita* was provided by the Laboratory of Nematology from the Federal University of Uberlândia and multiplied in pots with tomato ‘Santa Cruz’ and okra ‘Santa Cruz 47’ plants kept for 60 days in a greenhouse. After this period, the roots were processed using the nematode extraction technique described above with modifications. The roots were cut into 2-cm pieces and placed in a blender with a 0.5% sodium hypochlorite solution for 20 seconds, then poured into superimposed 100- and 500-mesh sieves (Hussey and Barker, 1973). The contents retained on the 500-mesh sieve were collected, and the suspension with *M. incognita* eggs was calibrated to the desired concentration.

*Screening for microorganisms with nematicidal potential:* To identify potential biocontrol agents and evaluate their nematicidal effects, 10 microorganisms from the CoMisBee collection were tested: five bacteria (Mq-MCK-07, Mq-NUT-16, Ms-MSA-9, Ms-MH-21, Ms-BDA2-41C) and five fungi (Fv-SAB-01, Mq-OAT-03, Ms-BDA2-35, Ms-ISP-46C, Ta-BDA2-26). The experiment was conducted in a greenhouse using a completely randomized design. For each treatment, five pots (1.5-L capacity) were prepared using a sand and clay substrate mixture (2:1 ratio), which had previously been analyzed to ensure the absence of nematodes.

For the bacterial treatments, 10 mL of bacterial culture that had been grown for 48 h was applied. For the fungal treatments, 10 mL of spore suspension, containing 3 × 107 spores, were used. Each treatment was applied directly to the planting furrow at the time of sowing soybean cultivar Brasmax Desafio RR 8473 RSF. Two seeds were sown per pot, and one was removed before nematode inoculation to ensure all pots contained plants at the same developmental stage.

For the negative control, pots were prepared with only soybean seeds, while the positive control consisted of pots treated with the commercial product Nemat (Ballagro, São Paulo, Brazil) — based on *Paecilomyces lilacinus* — applied at the manufacturer's recommended rate (600 g/ha).

Ten days after treatment, the 60 pots were inoculated with 10 mL of an *M. incognita* suspension containing 500 eggs/mL (5,000 eggs per pot). Sixty days post-inoculation, the root mass was measured, and nematodes (eggs and J2) were extracted from the roots using the previously-described extraction technique and quantified using a Peters chamber under an optical microscope.

*Validation of nematicidal potential:* Based on the results of the screening assay, the bacterial isolate Mq-MCK-07 was selected for further evaluation, and its nematicidal effect was validated in a second experiment. Four treatments were compared: (1) untreated control (soybean plants without nematode inoculation); (2) negative control (soybean plants inoculated with *M. incognita*); (3) Mq-MCK-07 treatment (plants inoculated with *M. incognita* and treated with Mq-MCK-07); and (4) positive control (plants inoculated with *M. incognita* and treated with Nemat).

For each treatment, 10 pots (1.5-L capacity) were prepared using the same sand and clay substrate mixture as in the previous assay. For the Mq-MCK-07 treatment, 10 mL of bacterial culture grown for 48 h were applied to the planting furrow at the time of sowing. The positive control was treated with Nemat — based on *Paecilomyces lilacinus* — applied at the manufacturer's recommended rate (600 g/ha). Two seeds of the soybean cultivar Brasmax Desafio RR 8473 RSF were sown per pot, and one seedling was removed prior to inoculation to ensure uniform plant development across treatments.

Ten days after sowing, the pots assigned to *M. incognita* treatments were inoculated with 10 mL of nematode suspension containing 500 eggs/mL (5,000 eggs per pot). After 60 days of inoculation, nematodes were extracted from the roots and quantified using a Peters chamber under an optical microscope. The nematicidal effect was evaluated by comparing the initial and final nematode populations.

*Egg-hatching inhibition test:* To evaluate the ability of the isolate Mq-MCK-07 to inhibit *M. incognita* egg hatching, an assay was conducted in a 24-well plate with four treatments: distilled water (negative control); Luria-Bertani (LB) medium; Mq-MCK-07 culture, grown for 48 hours; and the supernatant from Mq-MCK-07 culture, grown for 48 hours. Each treatment included five replicates, with each well containing 50 eggs. The plate was incubated at 28 °C for 72 hours, after which the number of hatched J2 and unhatched eggs was counted using an optical microscope (Saikia et al., 2013; Tian et al., 2022). The hatching rate was calculated using the formula: Hatching rate (%) = (Number of hatched J2 / Total number of eggs) × 100.

*In vitro nematicidal effect test:* The nematicidal effect of the isolate Mq-MCK-07 on *M. incognita* J2 was tested in a 24-well plate with four treatments: distilled water (negative control); Luria-Bertani (LB) medium; Mq-MCK-07 culture, grown for 48 hours; and the supernatant from Mq-MCK-07 culture, grown for 48 hours. Each treatment included five replicates, with each well containing 100 *M. incognita* J2. The plate was incubated at 28 °C for 24 hours and then evaluated under optical microscopy. Nematodes were considered dead when their bodies were straight and did not move when stimulated with 0.5 M NaOH (Saikia et al., 2013; Tian et al., 2022). The mortality rate was calculated using the formula: Mortality rate (%) = (Number of dead J2 / Total number of J2) × 100.

*DNA extraction:* Genomic DNA was extracted from a representative isolate of the Mq-MCK-07 strain. One biological sample of the isolate Mq-MCK-07 was cultured in BHI medium and incubated for 48 hours at 37 °C with 200 rpm agitation. After incubation, the bacterial culture was centrifuged at 14,000 rpm for 10 minutes at 25 °C, and the supernatant was discarded. Next, 1 mL of extraction buffer (100 mM Tris-HCl, 500 mM NaCl, 50 mM EDTA, and 0.4% SDS) was added to the bacterial pellet and vortexed for 15 seconds. Subsequently, 50 μL of proteinase K was added, and the samples were incubated at 56 °C for 30 minutes. After incubation, the samples were centrifuged at 14,000 rpm for 10 minutes at room temperature.

The supernatant was transferred to a new microtube containing 330 μL of isopropanol. The tubes were gently inverted 30 times and then centrifuged at 14,000 rpm for 10 minutes at room temperature. The supernatant was discarded, and 500 μL of 70% ethanol was added to the pellet. After centrifugation for 30 seconds, the supernatant was discarded; this step was repeated twice to ensure DNA purification. The supernatant was discarded again, and the DNA pellet was air-dried for 15 minutes. Finally, the pellet was re-suspended in 50 μL of ultra-pure water, and the quantity and quality of the extracted DNA were assessed using a Nanodrop 2000 Spectrophotometer (Thermo Fisher Scientific, Waltham, MA) and agarose gel electrophoresis.

*Genome sequencing and data analysis:* Whole-genome sequencing of the Mq-MCK-07 isolate was performed using the BGISEQ-500 sequencing system with DNBSEQ technology at BGI Genomics (Hong Kong, China). For DNBSEQ short-read library preparation, genomic DNA was fragmented and underwent size selection. The sequencing library had an insert size of 300 bp with a paired-end sequencing length of 150 bp. The selected fragments were 3′-adenylated through end-repair and adapter-ligation. A single-strand circular DNA was formed as the final library and quality control was performed using a clean FastQ file with a Phred+33 quality score threshold.

After sequencing, all raw data were trimmed and low-quality sequences were filtered using SOAPnuke v2.1.9 (Chen et al., 2018). Assembled contigs shorter than 150 bp, or containing an N content of 0.1% or higher, were discarded from subsequent analyses. The output read quality score system was set to Phred+33.

The whole genome was assembled using the SPAdes genome assembler v3.15.4 and compared against the NCBI database using the BLASTN tool. Species identification was carried out using an in-house algorithm (unpublished data). Prediction of regions associated with antimicrobial resistance or virulence genes was performed using ABRicate (Seemann, 2023). Secondary metabolite analysis was conducted with antiSMASH 7.0 software (Blin et al., 2023), and Artemis was used for genome visualization and annotation (Carver et al., 2012).

*Statistical analysis:* Data analysis was performed using Graphpad Prism software (version 10.1.1) using one-way analysis of variance (ANOVA) and Tukey multiple comparison tests (*p* < 0.05). All data are presented as mean ± standard deviation.

## Results

**Figure 1:**
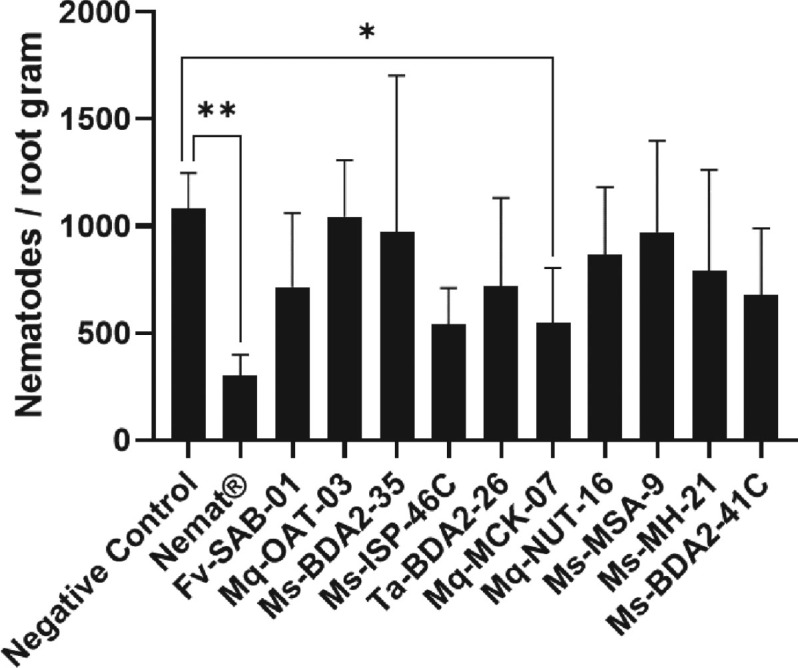
Nematicidal activity of microorganisms isolated from stingless bee larval food against *M. incognita* inoculated in pots with soybean. The negative control used water and the positive control used the commercial biocontrol product Nemat (recommended dosage). Data analysis was performed using One-Way analysis of variance (ANOVA) and Tukey multiple comparison tests in the Graphpad Prism software (version 10.1.1) (Dotmatics, Boston, MA). ^*^p < 0.05; ^*^^*^p < 0.01.

*Screening for microorganisms with nematicidal potential:* The evaluation of the nematicidal potential of 10 microorganisms tested from CoMisBee demonstrated that only the bacterial isolate Mq-MCK-07 exhibits a probable nematicidal effect, as it was able to reduce the number of nematodes per gram of soybean root when compared to the control treatment and other treatments (Fig. 1).

**Figure 2:**
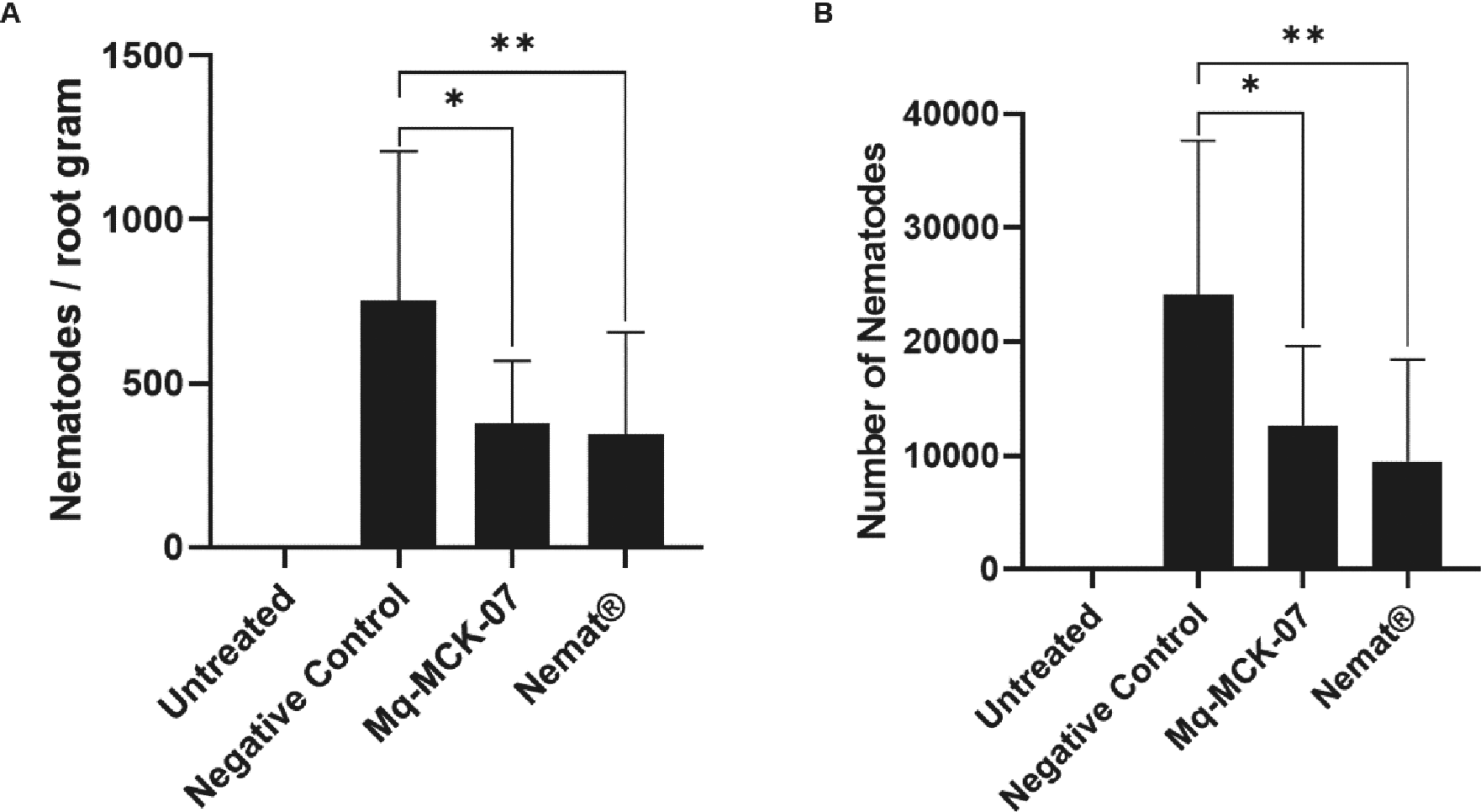
The effect of isolate Mq-MCK-07 on *M. incognita* inoculated in pots with soybean. (A) Number of nematodes per root gram 60 days after inoculation. (B) Number of nematodes in the soybean roots 60 days after inoculation. Untreated pots with no nematode inoculation were set to check possible contaminations among treatments. The negative control used water and the positive control used the commercial biocontrol product Nemat (recommended dosage). Data analysis was performed using One-Way analysis of variance (ANOVA) and Tukey multiple comparison tests in the Graphpad Prism software (version 10.1.1). ^*^p < 0.05; ^*^^*^p < 0.01.

*Validation of the nematicidal effect of Mq-MCK-07 on M. incognita:* In the validation assay, the bacterial isolate Mq-MCK-07 was efficient in reducing the total number of nematodes in soybean roots and in reducing the number of nematodes per gram of root when compared to the control treatment. This result validates its potential effect against *M. incognita*. It showed no significant difference when compared with the commercial treatment Nemat (Fig. 2).

**Figure 3:**
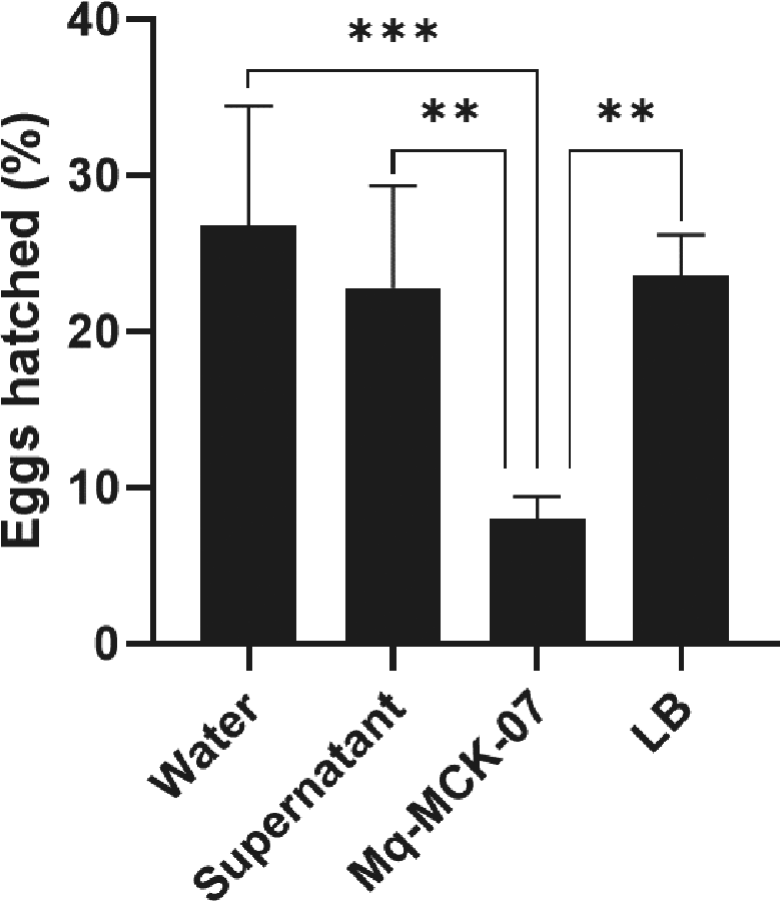
The Mq-MCK-07 bacteria inhibited *M. incognita* egg-hatching *in vitro*. Egg hatching rates were evaluated with 50 *M. incognita* eggs incubated at 28 °C for 72 h with different treatments. The negative control used distilled water. Data analysis was performed using One-Way analysis of variance (ANOVA) and Tukey multiple comparison tests in the Graphpad Prism software (version 10.1.1). ^*^^*^p < 0.01; ^*^^*^^*^p < 0.001.

*Inhibition of Meloidogyne incognita egg hatching:* The *M. incognita* egg-hatching rates after 72 h of incubation with different treatments were 26.8% in water (100% v/v, pH 6.8); 23.6% in Luria Bertani medium (50% v/v, pH 7.2); 22.8% in Mq-MCK-07 supernatant (50% v/v, pH 8.6); and 8% in culture medium with Mq-MCK-07 (50% v/v, pH 8.6), indicating that the presence of Mq-MCK-07 caused egg-hatching inhibition (Fig. 3).

**Figure 4:**
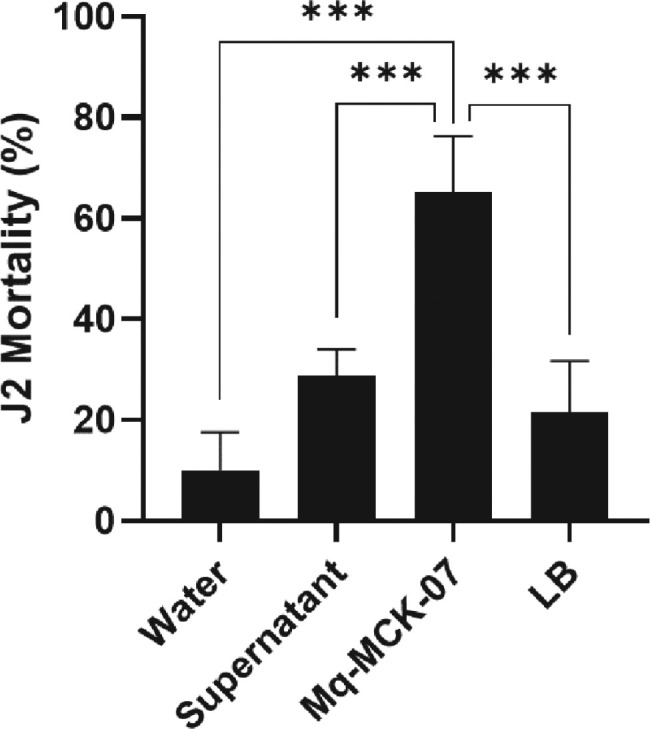
The isolate Mq-MCK-07 showed a nematicidal effect on J2 of *M. incognita* in the *in vitro* test. Mortality rates were evaluated with 100 J2 of *M. incognita* incubated at 28 °C for 24 h with different treatments. The negative control was distilled water. Data analysis was performed using One-Way analysis of variance (ANOVA) and Tukey multiple comparison tests in the Graphpad Prism software (version 10.1.1). ^*^^*^^*^p < 0.001.

*Nematicidal effect on juveniles (J2) of Meloidogyne incognita:* The mortality rates of *M. incognita* J2 after incubation with different treatments were 10% in water (100% v/v, pH 6.8); 21.6% in Luria Bertani medium (50% v/v, pH 7.2); 28.8% in Mq-MCK-07 supernatant (50% v/v, pH 8.6); and 65.2% in culture medium with Mq-MCK-07 (50% v/v, pH 8.6), indicating that the isolate Mq-MCK-07 had a nematicidal effect against J2 of *M. incognita* (Fig. 4).

**Figure 5:**
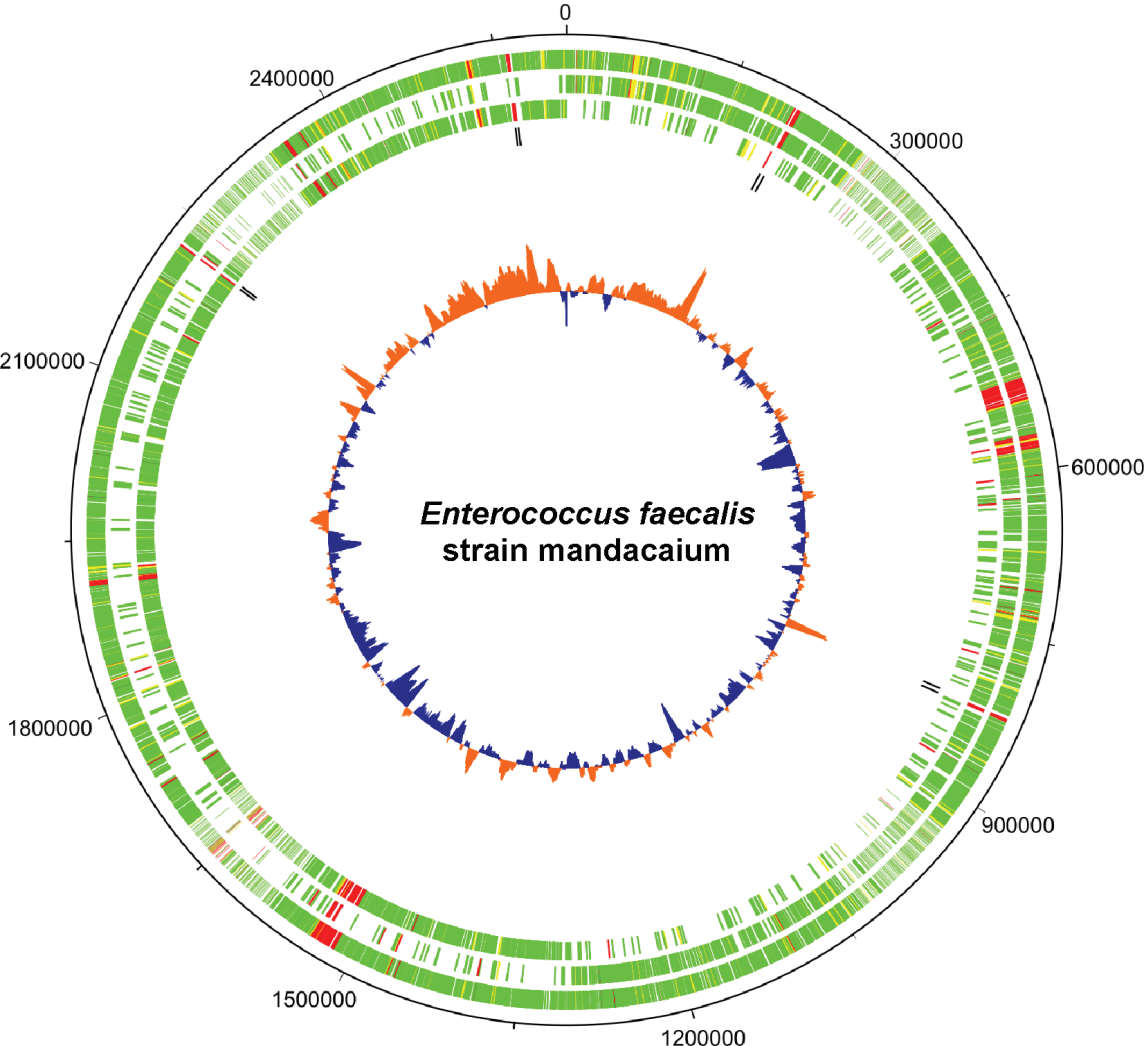
Graphical circular map of *Enterococcus faecalis* strain mandacaium chromosome (2.61 Mb). From outer circle to the center: all CDS on forward and reverse strands, all CDS on forward strand, all CDS on reverse strand, all rRNAs on forward and reverse strands. **Green**: > 95% of identity with reference genome; **Yellow**: 70% to 95% of identity with reference genome; **Red**: < 70% of identity with reference genome; **Black**: rRNA sequences. Inner circle shows GC content (**Blue**: Below average; **Orange**: above average). The map was generated using DNAPlotter from Artemis (Carver et al., 2012).

**Figure 6:**
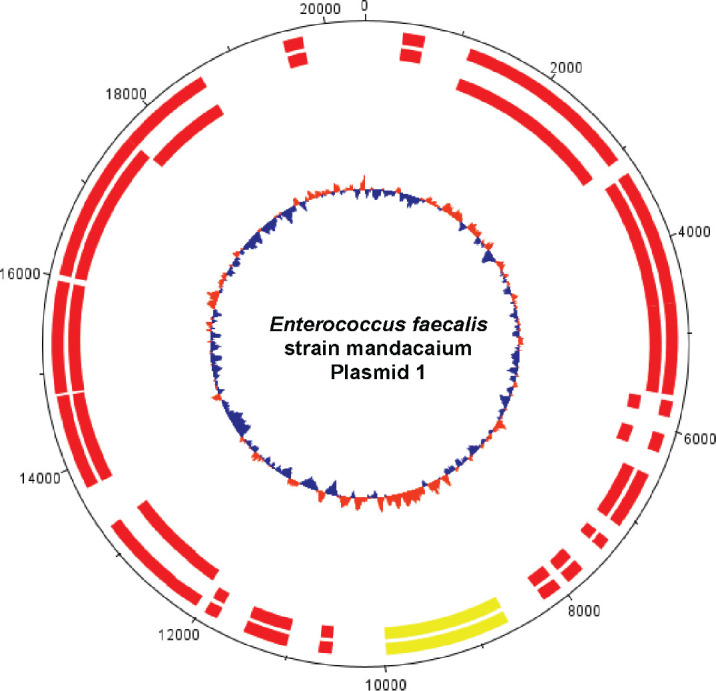
Graphical circular map of *Enterococcus faecalis* strain mandacaium plasmid 1 (20,406 bp). From outer circle to the center: all CDS on forward and reverse strands; all CDS on forward strand; all CDS on reverse strand. **Green**: > 95% of identity with reference genome; **Yellow**: 70% to 95% of identity with reference genome; **Red**: < 70% of identity with reference genome. Inner circle shows GC content (**Blue**: Below average; **Orange**: above average). The map was generated using DNAPlotter from Artemis (Carver et al., 2012).

**Figure 7:**
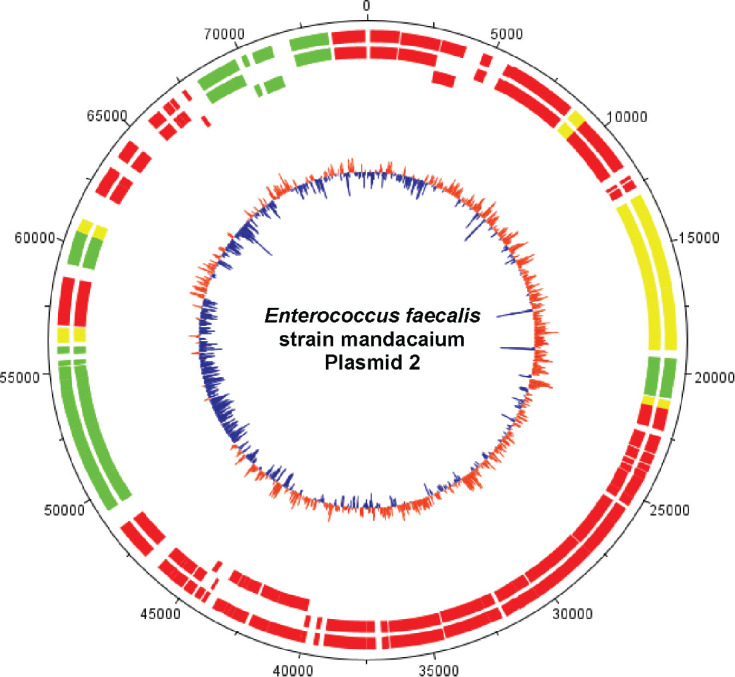
Graphical circular map of *Enterococcus faecalis* strain mandacaium plasmid 2 (74,988 bp). From outer circle to the center: all CDS on forward and reverse strands; all CDS on forward strand; all CDS on reverse strand. **Green**: > 95% of identity with reference genome; **Yellow**: 70% to 95% of identity with reference genome; **Red**: < 70% of identity with reference genome. Inner circle shows GC content (**Blue**: Below average; **Orange**: above average). The map was generated using DNAPlotter from Artemis (Carver et al., 2012).

*Species identification and genomic features:* The genome analysis with “Species Matching” indicated a match between the bacterial isolate Mq-MCK-07 and *Enterococcus faecalis* sequences deposited on the NCBI database. Due to the origin of this isolate of *Enterococcus faecalis* strain, we decided to name it mandacaium (*E. faecalis mandacaium*). Further analysis of genome characteristics revealed that the *E. faecalis mandacaium* genome comprises a circular chromosome of 2.6 Mb (Fig. 5), and it was deposited in NCBI with the accession number CP159511. The *E. faecalis mandacaium* chromosome included 2,612,373 bp, with an average GC content of 37.91%. There were 2,481 protein-coding genes found, of which two presented 95% or more identity with proteins found in the reference genome for the alignment. There were also 46 tRNAs and 8 rRNAs found, comprising a total of 2,535 genes. Two plasmid sequences were predicted in the analysis, named plasmid 1, with 20,406 bp (Fig. 6) and plasmid 2, with 74,988 bp (Fig. 7).

Using the ABRicate tool, possible regions encoding proteins for resistance to antimicrobial agents, such as vancomycin and clindamycin, were found in the genome, but none were found in the plasmid sequence. An antibiotic sensitivity test confirmed resistance to clindamycin, but vancomycin showed an antibiotic effect.

**Figure 8:**
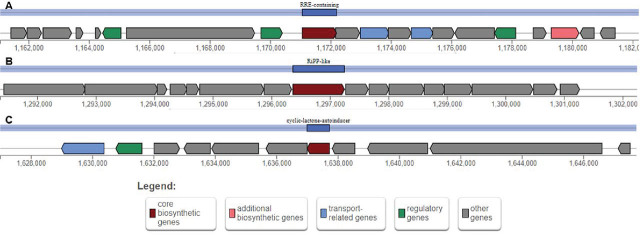
(A) RRE-containing region with a core biosynthetic gene encoding a sequence similar to the molecule Microcin 07 from *Escherichia coli*. (B) RiPP-like region with a core biosynthetic gene encoding a sequence similar to the Linocin M18. (C) Cyclic-lactone-autoinducer region with a core biosynthetic gene encoding a sequence similar to the molecule Enterocin NKR-5-3B from *Enterococcus faecium*.

The antiSMASH genome analysis tool detected three regions of secondary metabolite biosynthesis gene clusters in *E. faecalis mandacaium* (Fig. 8), encoding the bacteriocins Enterocin NKR-5-3B (100% similarity); Linocin M18 (94% similarity); Microcin C7 (100% similarity); and Linocin M18 (94% similarity).

**Figure 9:**
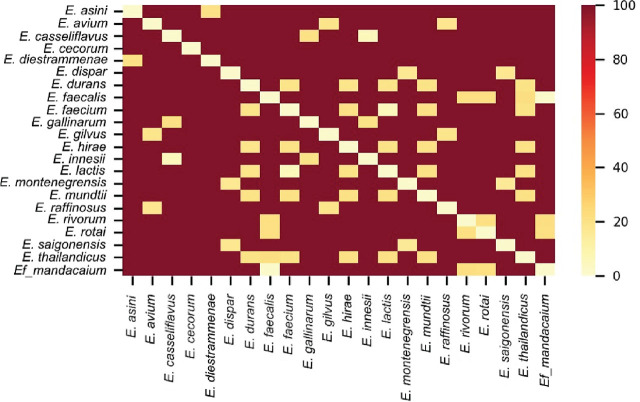
Heat map of the Average Nucleotide Identity (ANI) analysis performed with *Enterococcus faecalis* strain mandacaium and *Enterococcus* spp. genomes deposited in the NCBI database. The genomic distance was calculated on the scale of **100 ANI**. A distance of 0 (lightest color) corresponds to **100% ANI**, indicating maximum similarity between genomes, while a distance of **100** (darkest color) corresponds to **0% ANI**, indicating no similarity. Intermediate colors reflect proportional distances based on ANI values.

An Average Nucleotide Identity (ANI) analysis was performed using fastANI on *Enterococcus* spp. genomes deposited in the NCBI database. Only sequences with more than 2 million bases were selected to construct the matrix. The generated heat map confirmed a high degree of similarity between the genome of our isolate and the *E. faecalis* reference genome, though they were not identical (Fig. 9).

## Discussion

The use of chemical nematicides has been increasingly discouraged or even banned in several countries, due to the potential environmental and human harm these formulations can cause (Coyne et al., 2018; Abd-Elgawad, 2021). One of the most effective chemical nematicides in controlling *M. incognita*, abamectin, can pose risks to humans and the environment when used in high concentrations (Nasr et al., 2016; El-Marzoky et al., 2022).

The toxic effect of products used in the chemical control of pathogens on stingless bee species has been demonstrated in several recent studies (Aljedani, 2017; Li et al., 2022; Farder-Gomes et al., 2024; Obregon et al., 2024). The loss of biodiversity in these bees presents not only ecological but also economic problems, considering their importance in the pollination processes of crops of agronomic interest (Heard, 1999; Slaa et al., 2006; Atmowidi et al., 2022; Santos et al., 2022). The microbiota associated with stingless bees' larval food has also been shown to be an important source of organisms with numerous biotechnological applications, including potential biocontrol of pathogens (Santos et al., 2022).

In light of this, recent research has focused on more ecologically viable alternatives for controlling *M. incognita*, including the aforementioned biological control methods (Hu et al., 2022; Tian et al., 2022). In recent years, several studies have found microorganisms with specific activity against *M. incognita* (Kim et al., 2018; Park et al., 2020; Pocurull et al., 2020; Sharma et al., 2020; Wang et al., 2022). In this context, the present study evaluated the potential of microorganisms isolated from the larval food of stingless bees for the biocontrol of *M. incognita*.

Based on the results obtained from the bioassay conducted on soybean plants, among the ten microbial isolates evaluated, the bacteria Mq-MCK-07 — isolated from the stingless bee *Melipona quadrifasciata* (commonly known as mandaçaia) — exhibited the most significant nematicidal activity. This strain effectively reduced both the total nematode population in the soybean roots and the nematode density per gram of root tissue. After whole-genome sequencing, this bacteria was identified as *Enterococcus faecalis* strain mandacaium. To investigate its biocontrol mechanism, the microorganism and its supernatant were incubated with nematode eggs and juveniles, following protocols established in previous studies (Saikia et al., 2013; Tian et al., 2022). The tests demonstrated that the *E. faecalis* strain mandacaium was capable of inhibiting egg hatching and exhibited a nematicidal effect against *M. incognita* J2, with efficacy comparable to that of bacterial strains currently being used for this purpose, such as *Bacillus amyloliquefaciens*, *Bacillus megaterium*, and *Pseudomonas fluorescens* (Rani et al., 2022). Previous studies have demonstrated that *Bacillus* spp. can exhibit similar biocontrol effects through both bacterial applications and supernatant treatments (Hu et al., 2022; Tian et al., 2022). However, the supernatant of *E. faecalis* strain mandacaium did not show any significant effects on either egg hatching or J2 mortality. To date, no published reports have documented the use of *Enterococcus* spp. for the biological control of PPNs or other pathogens of agronomic importance.

The species *Enterococcus faecalis* is a Gram-positive bacteria that commonly lives in the guts of humans and animals. Under certain circumstances, depending on the strain, it can become mildly pathogenic to its host (Sifri et al., 2002; King et al., 2016). Like other *Enterococcus* species, it is a common source of hospital-acquired infections and can affect surgical sites, the bloodstream, and the urinary tract (Richards et al., 2000). The salience of the species in hospital environments is accentuated by its recognized intrinsic and acquired resistance to antibiotics (Murray, 1990; Rice, 2001).

In the genomic analysis of *E. faecalis* strain mandacaium, the presence of antibiotic-resistance genes was identified. Notably, the VanHBX gene, which confers resistance to vancomycin, was detected. This finding aligns with the well-documented resistance of *Enterococcus* spp. to this antibiotic (Uttley et al., 1988; Murray, 1990; Cetinkaya et al., 2000; Rice, 2001; Johnston and Jaykus, 2004; Aminov and Mackie, 2007; McInnes et al., 2024). The Lsa(A) gene was also found, which encodes an ATP-binding cassette (ABC) transporter and is recognized as an intrinsic resistance gene in *E. faecalis*, conferring resistance to clindamycin and quinupristin-dalfopristin (Singh et al., 2002; Sharkey et al., 2016). However, the phenotypic antibiotic susceptibility test revealed that *E. faecalis* mandacaium exhibited resistance to clindamycin, which was expected due to the presence of the Lsa(A) gene. However, it did not show resistance to vancomycin, despite harboring the VanHBX gene. Intrinsic resistance in *E. faecalis* is typically chromosomally encoded and not readily transferable between bacteria (Guan et al., 2024).

Interactions between *Enterococcus faecalis* and nematodes have typically been described in model organisms (King et al., 2016). Several studies indicate that *E. faecalis* is commonly found in both animal and human microbiomes, where it acts as either a commensal or a pathogenic agent (Sifri et al., 2002; Cruz et al., 2012; Kommineni et al., 2015). The bacteria can colonize the gut of *Caenorhabditis elegans* and act as a pathogenic agent, suppressing its population (Sifri et al., 2002; Sim and Hibberd, 2016). Multiple strains of *E. faecalis* and *E. faecium* have been shown to have nematicidal effects on *C. elegans* eggs and juveniles, with some strains of *E. faecalis* also killing *C. elegans* adults (Garsin et al., 2001). The anti-nematodic activity of *E. faecalis* has also been demonstrated against the mammalian parasitic nematode *Trichinella spiralis* (Schofs et al., 2014).

Interestingly, *E faecalis* can also protect its *C. elegans* host against infections from more virulent pathogens such as *Staphylococcus aureus*, crossing the parasitism-mutualism continuum (Chamberlain et al., 2014; King et al., 2016). Much of this protective trait can be attributed to the production of bacteriocins — antimicrobial peptides that are often encoded on pheromone-responsive plasmids in enterococcal colonization dynamics (Kommineni et al., 2015). Bacteriocins are ribosomally synthesized and post-translational modified peptides (RiPPs) with described antimicrobial activity that usually manifests in antagonistic activity towards microbial strains that are closely related to the producer strain. The bacteriocins can thus be used as natural food preservatives and therapeutic antibiotics (Arnison et al., 2013; Perez et al., 2016). Using the antiSMASH genome analysis tool, regions of secondary metabolite biosynthetic gene clusters of *E. faecalis mandacaium* were identified, encoding sequences with significant similarity to previously-described bacteriocin genes.

The bacteriocin Enterocin NKR-5-3B (Ent53B), which has a sequence with 100% similarity to that found in our analysis, is a circular bacteriocin originally found in *Enterococcus faecium* NKR-5-3 strain that has been described as having antimicrobial activity against Gram-positive bacteria such as *Bacillus* spp., *Lactococcus* spp. and *Listeria innocua* (Ishibashi et al., 2012; Perez et al., 2016; Wang et al., 2023). Many bacteriocins have been described in *E. faecalis* (Martín-Platero et al., 2006; Kommineni et al., 2015).

Bacteriocins from *Bacillus subtilis* and *Bacillus amyloliquefaciens* have been described as biocontrol agents for *M. incognita* (Migunova and Sasanelli, 2021). The borosins — another group of RiPPs commonly found in bacteria — have also been described as nematicidal agents. Omphalotins and the lentinulins are cyclic peptides from the borosin group with potent nematicidal activity against *M. incognita* (Mayer et al., 1997; Sterner et al., 1997; Ford et al., 2022; Imani et al., 2022; Zhong et al., 2023). In the initial analysis of *E. faecalis* strain mandacaium genome, however, none of the peptides found were described as having nematicidal activity. Due to this background, *E. faecalis* strain mandacaium mechanism of action in *M. incognita* biocontrol needs to be investigated in further analysis.

In summary, the *Enterococcus faecalis* strain mandacaium, isolated from stingless bees' larval food, exhibited nematicidal activity against *Meloidogyne incognita* both *in vitro* and when tested in soybean pots. Genome analysis revealed that this strain harbors several genes potentially involved in the biocontrol of various pathogens. This study therefore provides a theoretical basis for considering *E. faecalis* strain mandacaium's use as an eco-friendly nematicide.
